# Zooplankton and micronekton respond to climate fluctuations in the Amundsen Sea polynya, Antarctica

**DOI:** 10.1038/s41598-019-46423-1

**Published:** 2019-07-12

**Authors:** Hyoung Sul La, Keyhong Park, Anna Wåhlin, Kevin R. Arrigo, Dong Seon Kim, Eun Jin Yang, Angus Atkinson, Sophie Fielding, Jungho Im, Tae-Wan Kim, Hyoung Chul Shin, SangHoon Lee, Ho Kyung Ha

**Affiliations:** 1 Korea Polar Research Institute, Incheon, Korea; 2 Department of Marine Sciences, University of Gothenburg, Gothenburg, Sweden; 3 Department of Earth System Science, Stanford University, California, USA; 4 Korea Institute of Ocean Science and Technology, Busan, Korea; 5 Plymouth Marine Laboratory, Plymouth, United Kingdom; 6 British Antarctic Survey, Cambridge, United Kingdom; 7 Ulsan National Institute of Science and Technology, Ulsan, Korea; 8 Department of Ocean Sciences, Inha University, Incheon, Korea

**Keywords:** Climate-change impacts, Marine biology

## Abstract

The vertical migration of zooplankton and micronekton (hereafter ‘zooplankton’) has ramifications throughout the food web. Here, we present the first evidence that climate fluctuations affect the vertical migration of zooplankton in the Southern Ocean, based on multi-year acoustic backscatter data from one of the deep troughs in the Amundsen Sea, Antarctica. High net primary productivity (NPP) and the annual variation in seasonal ice cover make the Amundsen Sea coastal polynya an ideal site in which to examine how zooplankton behavior responds to climate fluctuations. Our observations show that the timing of the seasonal vertical migration and abundance of zooplankton in the seasonally varying sea ice is correlated with the Southern Annular Mode (SAM) and El Niño Southern Oscillation (ENSO). Zooplankton in this region migrate seasonally and overwinter at depth, returning to the surface in spring. During +SAM/La Niña periods, the at-depth overwintering period is shorter compared to −SAM/El Niño periods, and return to the surface layers starts earlier in the year. These differences may result from the higher sea ice cover and decreased NPP during +SAM/La Niña periods. This observation points to a new link between global climate fluctuations and the polar marine food web.

## Introduction

Zooplankton are an essential link between primary producers and higher trophic levels. The vertical migration of zooplankton moves a massive biomass within the water column with impacts on trophic interactions and biogeochemical cycles^1,2^. Zooplankton feed on primary producers, repackaging organic matter into rapidly sinking fecal pellets, and their vertical migration can be an important mechanism for carbon export and sequestration to depth^3^. Active vertical migration of zooplankton could contribute up to a 14% increase in carbon sinking from the euphotic zone into deeper water^4^.

Many polar zooplankton, including the large biomass-dominant copepods, chaetognaths, salps, key euphausiid species and possibly pteropods, undertake seasonal vertical migration^5–8^. They actively feed and reproduce in spring and summer and migrate to deeper water during autumn in the preparation for overwintering, and remain in deep water during the long winter period. However, the factors driving the extent and phenology of these vertical migrations are still poorly understood. In particular, our understanding of how they respond to climate fluctuations is lacking, mostly due to the lack of high-resolution time series in challenging polar environments.

Rapid climate change has been shown to drive significant physical and ecological changes^9–12^. In the Southern Ocean, these changes include ocean warming^13^, glacial melt and retreat^14^, and sea ice loss^15^. These alterations of the marine environment impact phytoplankton^16–18^, zooplankton^19–22^, fish, and penguins^23,24^, although the relative roles of climate and overfishing have complicated the interpretation of higher predator responses. The region’s annual variability of phytoplankton biomass and sea ice concentration (SIC) is closely related to climate shifts^25,26^. ENSO and SAM are significant drivers of the trend of SIC and thereby help to control the conditions for phytoplankton growth. The Amundsen Sea is located in one of the most rapidly warming regions on Earth^27^. This region is presently undergoing a rapid reduction in sea ice^16^ and retreat and thinning of glaciers^28,29^ and harbors one of the most productive coastal polynyas (per unit area) in the Southern Ocean^30^.

Here, we present results obtained from satellite remote sensing (surface solar radiation (SSR), SIC and NPP) and subsurface moored instrumentation (acoustic backscatter and sediment traps) from 2010 to 2013 (see Methods for a detailed description of the measurements). These results represent the longest existing continuous record of acoustic backscatter from a highly productive polynya, coinciding with a period of cooling and heavy sea ice years.

## Results

**Figure 1:**
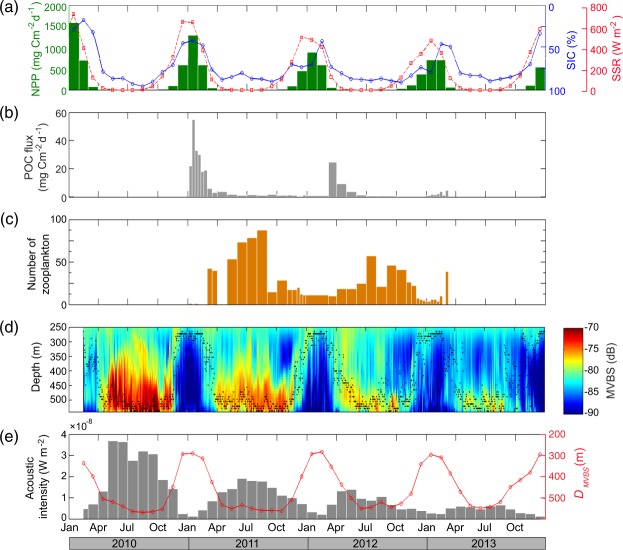
Multi-year ocean mooring data and related time series. **(a**) Net primary production (NPP), sea ice concentration (SIC), and surface solar radiation (SSR). (**b**) POC flux measured at the sediment trap of the K1 mooring. (**c**) Seasonal changes in the total number of intact post-larval zooplankton in the sediment trap from January 2011 to March 2013. (**d**) Mean volume backscatter strength (MVBS) measured at the S1 mooring. Black dots indicate the daily mean depth of the maximum MVBS (*D*_*MVBS*_). (**e**) Monthly means of acoustic intensity (grey bar) and *D*_*MVBS*_ (red line).

During our study, the Amundsen Sea shelf area had a seasonal ice cover with an expanding polynya from January to March (Fig. 1 and Supplementary Fig. S1). Between 2010 and 2013, the mean NPP in the area peaked in January (Supplementary Fig. S2), co-varying with the SIC and SSR (Fig. 1a). The mean NPP (nearly all taking place during summer) decreased from 789 to 493 mg C m^−2^ d^−1^ between 2010 and 2013. During the same period, the mean SIC increased by 15%. The interannual variation in SIC was strongly correlated to the summertime NPP (*r* = −0.73, *p* < 0.05) and the annual NPP peak as expected^31^ coincided with the SIC minimum. This implies that the intense phytoplankton bloom is triggered by the increase in open water area (Supplementary Fig. S3).

Particulate organic carbon (POC) fluxes obtained from the moored sediment trap at about 400 m show large seasonal and interannual variations, with maxima coinciding with the SIC minima (Fig. 1a,b). Over 90% of the annual POC flux was generated between January and March, with peaks in January 2011 (54.9 mg C m^−2^ d^−1^) and March 2012 (24.8 mg C m^−2^ d^−1^). A rapid decrease in POC flux in mid-March 2011 coincided with a rapid decrease in NPP at the surface. The high POC flux in summer 2010/2011 coincided with high NPP, while the summer 2012/2013 had lower NPP and lower POC flux (Fig. 1b). Ungrazed phytoplankton (mostly diatoms) comprised over 75% of POC flux during summer. Observations of POC content and stable nitrogen isotope analysis (Supplementary Fig. S4) point to a shift in sinking material from fresh phytoplankton in summer to zooplankton carcasses in winter. The abundance of intact zooplankton in the sediment trap also showed a seasonal cycle similar to that of POC (Fig. 1c), peaking between autumn and spring and being lower during summer. The zooplankton population was dominated by copepods, amphipods, ctenophores, ostracods, and pteropods, which constituted about 70% to the total zooplankton swimmers (Supplementary Fig. S4). During summer, the abundance and vertical migration of these swimmers followed trends in sea ice melting and NPP (Fig. 1). The monthly mean acoustic intensity in the deep scattering layers (DSLs) exhibited a strong positive correlation with intact zooplankton abundance (*r* = 0.57, *p* < 0.01) (Fig. 1c,e).

The DSL is a concentrated layer of scatterers comprising an assemblage of zooplankton (e.g. copepods, amphipods and small euphausiids) and micronekton (e.g. large euphausiids and small fish). The monthly mean acoustic intensity and the depth of the maximum acoustic intensity (Fig. 1e) provide information on the seasonality and interannual changes in zooplankton abundance and the depth of the primary habitat. During high NPP/low SIC years (2010 and 2011), the downward movement of zooplankton began as the phytoplankton bloom ended around February, and they migrated to the near-bottom (~540 m) in April. The zooplankton stayed below 450 m for 212 and 232 days in 2010 and 2011, respectively, during which time the surface was ice covered. During low NPP/high SIC years (2012 and 2013), however, the times spent at depth were much shorter (85 and 125 days in 2012 and 2013, respectively) than those in the previous two years. In addition, the depth of the maximum acoustic intensity during winter was shallower in 2013 (465 m) than in 2010 (520 m). The mean overwintering depth between July and October was linearly related to mean NPP during summer from December to February between 2010 and 2013 (overwintering depth = 0.12 NPP + 407, *r*^2^ = 0.89).

**Figure 2:**
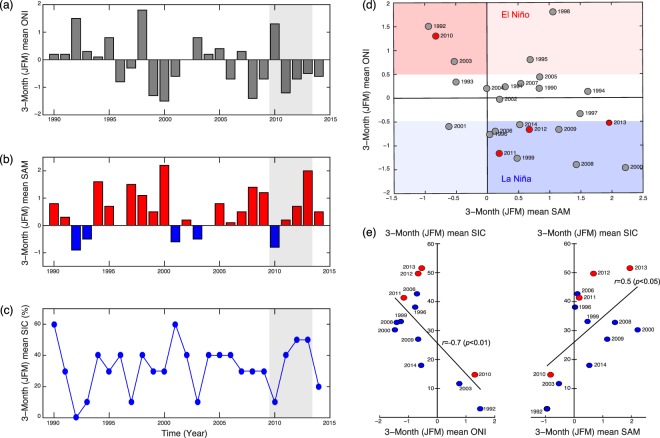
Temporal variability of the Southern Annular Mode (SAM) and El Niño Southern Oscillation (ENSO). (**a**) Mean Oceanic Niño Index (ONI). (**b**) SAM. (**c**) SIC during the summer (from January to March) in the period 1990 to 2014. (**d**) The relationship between the ONI and SAM in the period 1990 to 2014. (**e**) The relationship between the SIC and ONI (or SAM) during in-phase periods (+SAM/La Niña and −SAM/El Niño). The shaded areas in (**a–c)** indicate the mooring campaign periods.

Sea ice provides an essential ecological habitat and serves as an indicator of climate change^15^ and a driver of numerous ecosystem responses. The change in SIC during summer is linked to the atmospheric circulation and the climate indices of SAM and ENSO^32^. The high-latitude atmospheric variability is reinforced when the two indices are in phase (+SAM/La Niña or −SAM/El Niño), and thus their impact on the change in sea ice extent becomes more obvious during in-phase periods^33^. The 3-month (January to March) mean SIC in the Amundsen Sea coastal polynya exhibited a significant positive correlation with SAM (*r* = 0.5, *p* < 0.05) and a negative correlation with ENSO (*r* = −0.7, *p* < 0.01) during the in-phase periods of the last two decades (Fig. 2). This pattern was also evident during our observation period: the SIC during −SAM/El Niño (2010) was 30% lower than that during +SAM/La Niña (2013). This finding is consistent with the general high-latitude ice-atmosphere response to climate fluctuations^33^.

## Discussion

**Figure 3:**
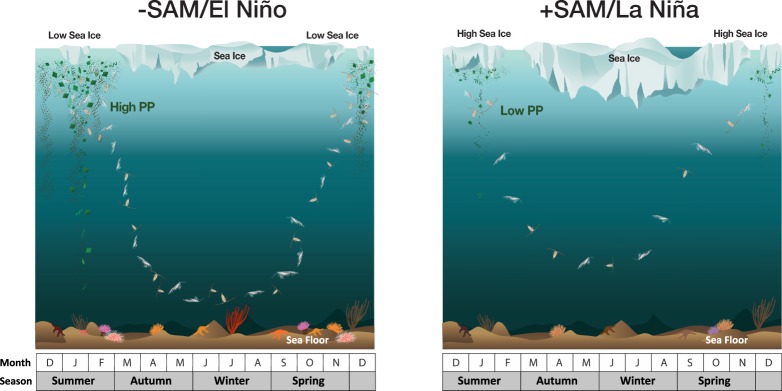
Effects of climate forcing on biological processes in the Amundsen Sea coastal polynya under two distinct climate regimes. The schematic illustration explains how climate fluctuations affect the phytoplankton bloom and SIC that control the seasonal vertical migration of zooplankton. Zooplankton remain longer near the bottom beginning to ascend in October when the summer SIC is low with high PP under −SAM/El Niño; in August, they begin to ascend to the surface when the summer SIC is high with low PP under +SAM/La Niña. The overwintering depth is shallower during the high summer SIC and low PP under +SAM/La Niña.

In highly seasonal polar environments, large-scale climatic modes can drive more local environmental conditions, whose effects propagate through phytoplankton to impact on zooplankton^25^. We have found that climatic variability, ENSO and SAM, could impact the zooplankton prey (phytoplankton biomass) and their habitat condition through variations in sea ice coverage in the Amundsen Sea during summer (Fig. 2 and Supplementary Fig. S3). During 2013 (+SAM/La Niña), high SIC corresponded with low phytoplankton, whereas during 2010 (−SAM/El Niño), low SIC was linked to an intense bloom. Likewise, zooplankton showed contrasting biomass and vertical behaviors during these two distinctly different environmental conditions, with higher biomass and longer overwintering period during −SAM/El Niño (Fig. 3). Studies in the Southern Ocean are increasingly finding links between climate, SIC, phytoplankton and zooplankton, but the mechanisms are region-specific and change over decadal time-scales^34^. While our study has too few years to draw definitive conclusions over the large-scale climate connections, it shows an important process that the local climate directly influences the distribution and seasonal migration of zooplankton. The following discussion develops this to provide a working hypothesis for how this system may be operating and guide design of longer-term observing systems.

During the 4-year study period, there was high interannual variability in acoustic intensity, SIC, NPP, POC flux, as well as climate indices. In the year of maximum SIC and high positive SAM (2013), SIC was high and NPP, acoustic intensity, and POC flux were all low. The acoustic backscatter results presented here show that zooplankton migrate to the upper water to feed or reproduce during the summer, and that the summertime NPP influences zooplankton abundance and the seasonal vertical migration during the rest of the year (Fig. 1d). The annual mean acoustic intensity implied by zooplankton abundance declined over the entire period, correlating with the trend of the annual mean NPP (*r* = 0.97, *p* < 0.05) and summertime SIC (*r* = −0.95, *p* < 0.05). One reason may be that the zooplankton during low NPP/high SIC years could not accumulate the large lipid stores needed to overwinter and diapause in deep waters^35^. Another explanation may be that the overall abundance and the size-spectrum of zooplankton were reduced and the taxa surviving the poor phytoplankton years had a migration strategy that enabled them to move up early to forage. The phytoplankton community composition and abundance of zooplankton have been observed to adjust to sea-ice seasonality in other regions^16^.

Many of the biomass dominant taxa, including copepods and euphausiids, feed and reproduce in the upper water column during the phytoplankton bloom, descending to depth in autumn and winter. With a single frequency ADCP, it is hard to identify which species is responsible for the acoustic backscatter, and taxa collected in the sediment traps may not provide a representative sample of the suite of living organisms resident in the polynya. However, a strength of our acoustic data is that it can provide long time series of the bulk zooplankton abundance and behavior at high resolution^8,36^. Our sampling in the summer^37^, when netting techniques are available to us, shows a high biomass of *E. crystallorophias* as well as *E. superba* in this region. While vertical migrations of the former are poorly known, the latter is known to perform strong seasonal vertical migrations to the seabed in shelf habitats^38,39^. Likewise, many of the large biomass-dominant copepods and salps perform extensive seasonal migrations^5,7^. Whatever the composition of zooplankton in these migrating DSLs, they represent an important but highly variable downward transport of carbon. At depth they survive by undergoing diapause or feeding opportunistically^40^, with both swimmers and carcasses entering the sediment trap. Respiration by these overwintering zooplankton provides a source of carbon to deep waters. For example, about half of the carbon sequestration in the north Atlantic has been estimated to derive from migrating zooplankton^3^.

Climate change could influence population and community dynamics of zooplankton through the change of physical, chemical, and biological environments. However, the implications of climatic change on the seasonal vertical migration behavior of zooplankton under sea ice are seldom considered. Our results show that these migrations of zooplankton are modulated in response to climate fluctuations (SAM/ENSO) over the Amundsen Sea coastal polynya, potentially even synchronizing the life cycles of zooplankton with the seasonal variability of NPP and SIC (Fig. 3). The interaction between climate and sea ice observed over the measurement period is consistent over the past two decades (Fig. 2) in the Amundsen Sea coastal polynya. This interaction implies that zooplankton in productive coastal polynyas in the Southern Ocean adjust their behavior in response to climate fluctuations with ramifications for carbon export in a changing climate.

## Methods

The time series data from two moorings in the western part of the outer Amundsen Sea shelf was used (Supplementary Fig. S6). The study site is one of the deep troughs in the Amundsen Sea, where warm water has been observed to flow towards the continent^32,33^.

The S1 mooring was located at 72° 27.35′S, 116° 20.33′W, and consisted of a 150-kHz acoustic Doppler current profiler (ADCP) (RDI, WorkHorse Quartermaster) and five MicroCats (Seabird, SBE-37). This mooring was located near the center of the pack ice zone in the Dotson Trough, where a deep current transports warm, salty water towards the ice shelves farther south^41,42^. The mooring was deployed in February 2010 and serviced and re-deployed in December 2010 and March 2012 and was successfully recovered in January 2014 (Supplementary Table S1). The five MicroCats were distributed between depths of 540 and 320 m, where they measured conductivity, temperature, and pressure. The ADCP was mounted at the bottom of the mooring at a depth of approximately 556 m. Its configuration was upward-looking using four beams with a beam angle of 20°, and it measured current velocity and acoustic backscatter between 540 and 250 m. The number of depth cells was 44 with a bin size of 8 m. The sampling interval was set to 5 pings per ensemble every 15 min with evenly distributed pings. The mooring was stable, with less than 1° in pitch/role angle during the observation period. The acoustic backscatter recorded by the ADCP was converted to mean volume backscattering strength (MVBS, dB *re* 1 m^−1^) using the sonar equation presented by Deines^43^.

The K1 mooring was located at 72° 23.21′S, 117° 46.63′W, and consisted of two MicroCats and a time-series sediment trap (McLane, PARFLUX Mark 7G, aperture diameter = 80 cm and height/diameter = 2.5) at 414 m depth. The mooring was deployed at a depth of 530 m approximately 50 km northwest of S1 from December 2010 to January 2014.

From the sediment trap, we obtained a time series of POC flux, contents of biogenic and non-biogenic components, and zooplankton composition in swimmers. The cup opening time and intervals are shown in Supplementary Table S2. After recovery, the sampling bottles were filled with a mixture of seawater and 5% formalin buffered with sodium borate and then stored in a refrigerator at 4 °C for further processing. Whole trap samples were split with a rotary splitter, and one-fifth of each sample was used to estimate POC flux. All visible intact zooplankton were removed prior to sample processing to estimate POC flux.

Daily net primary production (mg C m^−2^ d^−1^) was calculated between 71–74.5°S and 120–110°W on the basis of satellite-derived Chl-*a*, sea surface temperature, and SIC, with estimates of mixed-layer depth, cloud cover, and spatial irradiance derived according to Arrigo *et al*.^44^.

The Special Sensor Microwave Imager/Sounder (SSMIS) SIC product on a 10 km Polar Stereographic Grid, which is produced by the EUMETSAT Ocean & Sea Ice Satellite Application Facility (OSI SAF), was used to identify the spatiotemporal distributions of SIC between 72–74.5°S and 120–110°W. These data are based on atmospherically corrected SSMIS brightness temperatures at 85 GHz and generated through the adaptive combination of bootstrap frequency mode (little weight at high SIC) and Bristol (little weight at low SIC) algorithms^45^. The SSR (W m^−2^) was retrieved at 12 h intervals between 72–74.5°S and 120–110°W from the European Centre for Medium Range Weather Forecasts (ECMWF) ERA-Interim reanalysis. The monthly SAM index was downloaded from the BAS website (http://www.nerc-bas.ac.uk/icd/gjma/sam.html), which is based on the methodology given in Marshall^46^. The Oceanic Niño Index (ONI) was obtained from the Climate Prediction Center (CPC, http://www.cpc.ncep.noaa.gov).

## Supplementary information

Supplementary Materials

## Data Availability

Raw and processed data for individual cruises, along with details of the processing, can also be obtained upon reasonable request to the first author (hsla@kopri.re.kr).

## References

[1] Neill WE (1990). Induced vertical migration in copepods as a defence against invertebrate predation. Nature.

[2] Tarling GA, Johnson ML (2006). Satiation gives krill that sinking feeling. Curr. Biol..

[3] Jónasdóttir SH, Visser AW, Richardson K, Heath MR (2015). Seasonal copepod lipid pump promotes carbon sequestration in the deep north Atlantic. Proc. Natl. Acad. Sci. USA.

[4] Archibald KM, David AS, Scott CD (2019). Modeling the impact of zooplankton diel vertical migration on the carbon export flux of the biological pump. Global Biogeochem. Cy..

[5] Atkinson A (2012). An overview of Southern Ocean zooplankton data: abundance, biomass, feeding and functional relationships. CCAMLR Sci..

[6] Conover RJ, Huntley M (1991). Copepods in ice-covered seas—distribution adaptations to seasonally limited food metabolism growth patterns and life cycle strategies in polar seas. J. Marine Syst..

[7] Marrari M, Daly KL, Timonin A, Semenova T (2011). The zooplankton of Marguerite Bay, western Antarctic Peninsula—Part I: abundance, distribution, and population response to variability in environmental conditions. Deep Sea Res. Part 2 Top. Stud. Oceanogr..

[8] La HS (2015). Acoustic backscatter observations with implications for seasonal and vertical migrations of zooplankton and nekton in the Amundsen shelf (Antarctica). Estuar. Coast. Shelf Sci..

[9] Walther GR (2002). Ecological responses to recent climate change. Nature.

[10] Edwards M, Richardson AJ (2004). Impact of climate change on marine pelagic phenology and trophic mismatch. Nature.

[11] Hoegh-Guldberg O, Bruno JF (2010). The impact of climate change on the world’s marine ecosystems. Science.

[12] Parmesan C, Yohe G (2003). A globally coherent fingerprint of climate change impacts across natural systems. Nature.

[13] Schmidtko S, Heywood KJ, Thompson AF, Aoki S (2014). Multidecadal warming of Antarctic waters. Science.

[14] Dutrieux P (2014). Strong sensitivity of Pine Island ice-shelf melting to climatic variability. Science.

[15] Stammerjohn S, Massom R, Rind D, Martinson D (2012). Regions of rapid sea ice change: an inter-hemispheric seasonal comparison. Geophys. Res. Lett..

[16] Montes-Hugo M (2009). Recent changes in phytoplankton communities associated with rapid regional climate change along the western Antarctic Peninsula. Science.

[17] Quinn PK, Bates TS (2011). The case against climate regulation via oceanic phytoplankton sulphur emissions. Nature.

[18] Arrigo KR, van Dijken GL, Strong AL (2015). Environmental controls of marine productivity hot spots around Antarctica. J. Geophys. Res. Oceans.

[19] Loeb V (1997). Effects of sea-ice extent and krill or salp dominance on the Antarctic food web. Nature.

[20] Rhode SC, Pawlowski M, Tollrian R (2001). The impact of ultraviolet radiation on the vertical distribution of zooplankton of the genus daphnia. Nature.

[21] Atkinson A, Siegel V, Pakhomov E, Rothery P (2004). Long-term decline in krill stock and increase in salps within the Southern Ocean. Nature.

[22] Bednaršek N (2012). Extensive dissolution of live pteropods in the Southern Ocean. Nat. Geosci..

[23] Barbraud C, Weimerskirch H (2001). Emperor penguins and climate change. Nature.

[24] Bost, C. A. *et al*. Large-scale climatic anomalies affect marine predator foraging behavior and demography. *Nat. Commun*. **6**, 10.1038/ncomms9220 (2015). 10.1038/ncomms9220 PMC4639794 26506134

[25] Saba GK (2014). Winter and spring controls on the summer food web of the coastal West Antarctic Peninsula. Nat. Commun..

[26] Matear RJ (2015). Sources of heterogeneous variability and trends in Antarctic sea-ice. Nat. Commun..

[27] Bromwich DH (2013). Central west Antarctica among the most rapidly warming regions on Earth. Nat. Geosci..

[28] Pritchard HD (2012). Antarctic ice-sheet loss driven by basal melting of ice shelves. Nature.

[29] Paolo FS, Fricker HA, Padman L (2015). Ice sheets. Volume loss from Antarctic ice shelves is accelerating. Science.

[30] Arrigo KR, van Dijken GL (2003). Phytoplankton dynamics within 37 Antarctic coastal polynya systems. J. Geophys. Res..

[31] Arrigo KR, Lowry KE, van Dijken GL (2012). Annual changes in sea ice and phytoplankton in polynyas of the Amundsen Sea, Antarctica. Deep Sea Res. Part 2 Top. Stud. Oceanogr..

[32] Goosse H, Lefebvre W, de Montety A, Crespin E, Orsi AH (2009). Consistent past half-century trends in the atmosphere, the sea ice and the ocean at high southern latitudes. Clim. Dyn..

[33] Stammerjohn SE, Martinson DG, Smith RC, Yuan X, Rind D (2008). Trends in Antarctic annual sea ice retreat and advance and their relation to el Niño–Southern oscillation and southern annular mode variability. J. Geophys. Res..

[34] Atkinson A (2019). Krill (Euphausia superba) distribution contracts southward during rapid regional warming. Nat. Clim. Change..

[35] Lee R, Hagen W, Kattner G (2006). Lipid storage in marine zooplankton. Mar. Ecol. Prog. Ser..

[36] Cisewski B, Strass VH (2016). Acoustic insights into the zooplankton dynamics of the eastern Weddell Sea. Prog. Oceanogr..

[37] La HS (2015). High density of ice krill (Euphausia crystallorophias) in the Amundsen sea coastal polynya, Antarctica. Deep Sea Res. Part I: Oceanogr. Res. Papers.

[38] Cleary AC, Durbin EG, Casas MC (2018). Feeding by Antarctic krill Euphausia superba in the West Antarctic Peninsula: differences between fjords and open waters. Mar. Ecol. Prog. Ser..

[39] Kane MK, Yopak R, Roman C, Menden-Deuer S (2018). Krill motion in the Southern Ocean: quantifying *in situ* krill movement behaviors and distributions during the late austral autumn and spring. Limnol. Oceanogr..

[40] Atkinson A (2002). Feeding and energy budgets of Antarctic krill Euphausia superba at the onset of winter-II. Juveniles and adults. Limnol. Oceanogr..

[41] Wåhlin AK (2013). Variability of warm deep water inflow in a submarine trough on the Amundsen Sea shelf. J. Phys. Oceanogr..

[42] Ha HK (2014). Circulation and modification of warm deep water on the central Amundsen shelf. J. Phys. Oceanogr..

[43] Deines, K. L. Backscatter estimation using broadband acoustic Doppler Current profilers, *Proceedings of the 6th IEEE Working Conference on Current Measurement*, San Diego, CA, 249–253, 10.1109/ccm.1999.755249 (1999).

[44] Arrigo KR (2008). Primary production in the Southern Ocean, 1997–2006. J. Geophys. Res..

[45] Eastwood, S. Sea ice product user’s manual OSI-401-a, OSI-402-a, OSI-403-a, version 3.11 Osi SAF (2014).

[46] Marshall GJ (2003). Trends in the southern annular mode from observations and reanalyses. J. Clim..

