# Supplementary material for: Zooplankton and micronekton respond to climate fluctuations in the Amundsen Sea polynya, Antarctica
Source: Sci Rep. 2019 Jul 12;9:10087. doi: 10.1038/s41598-019-46423-1 (PMC6626131; doi:10.1038/s41598-019-46423-1)
Supplement: Supplementary file 1 — Supplementary Materials [file 41598_2019_46423_MOESM1_ESM.pdf]

[Supplementary material]

Zooplankton and micronekton respond to climate fluctuations in the Amundsen Sea polynya, Antarctica

Hyoung Sul La[1], Keyhong Park[1], Anna Wåhlin[2], Kevin R. Arrigo[3], Dong Seon Kim[4], Eun Jin Yang[1], Angus Atkinson[5], Sophie Fielding[6], Jungho Im[7], Tae-Wan Kim[1], Hyoung Chul Shin[1], SangHoon Lee[1], and Ho Kyung Ha[1,8*]

[1]Korea Polar Research Institute, Incheon, Korea

[2]Department of Marine Sciences, University of Gothenburg, Gothenburg, Sweden

[3]Department of Earth System Science, Stanford University, California, USA

[4]Korea Institute of Ocean Science and Technology, Busan, Korea

[5]Plymouth Marine Laboratory, Plymouth, United Kingdom

[6]British Antarctic Survey, Cambridge, United Kingdom

[7]Ulsan National Institute of Science and Technology, Ulsan, Korea

[8]Department of Ocean Sciences, Inha University, Incheon, Korea

[*]Corresponding author

Tel: +82-32-860-7702; Fax: +82-32-862-5236

E-mail addresses: hahk@inha.ac.kr (H.K. Ha)

This supplementary material notes the location of the observations in the Amundsen Sea, Antarctica, where surface water is covered by seasonally varying sea ice; provides additional information about the seasonal variability of primary production during the observation period; and presents additional details on the zooplankton taxa caught by the sediment trap.

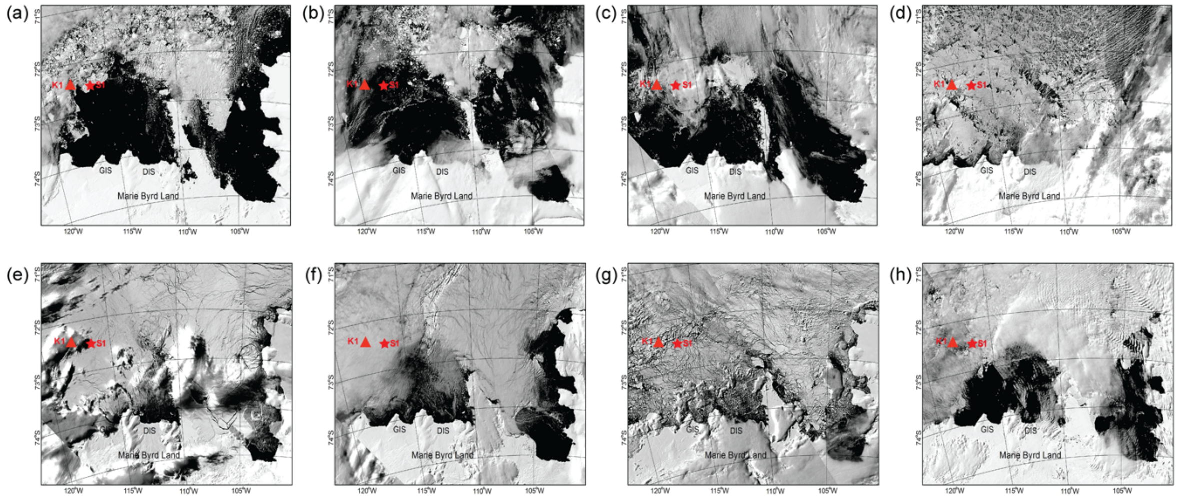

Figure S1. Discrete time series of the sea ice distribution captured by Moderate Resolution Imaging Spectroradiometer (MODIS) on NASA's Aqua satellite. a, January 3, **b,** February 4, **c,** March 3, **d,** April 5, **e,** September 18, **f,** October 19, **g,** November 8, and **h,** December 31 in 2010. The triangle (K1) and star (S1) indicate the respective mooring locations of the sediment trap and acoustic Doppler current profiler (ADCP). GIS: Getz Ice Shelf; DIS: Dotson Ice Shelf. Coastal polynyas are open water areas in the middle of ice-covered seas showing different seasonal and interannual variations in the periods of opening and closing. The Amundsen Sea coastal polynya between 72−74.5°S and 120−110°W is surrounded by the pack ice and two ice shelves (GIS and DIS); it is ice free due to increasing temperatures during the austral spring and summer and is ice covered during winter. In the Antarctic coastal polynyas, sea ice production is greatest at the beginning stage of freezing during early winter (March and April), and thin sea ice can form on the polynyas during the freezing period[47]. The sea-ice cover around the mooring locations is visually much smaller from January to March than in the other months, showing a similar trend to the SIC over the 13 years between 1997 and 2010[31]. The MODIS data was obtained from the LANCE-MODIS data system (https://lance.modaps.eosdis.nasa.gov/) and visualized with ArcGIS 10.3 (www.arcgis.com).

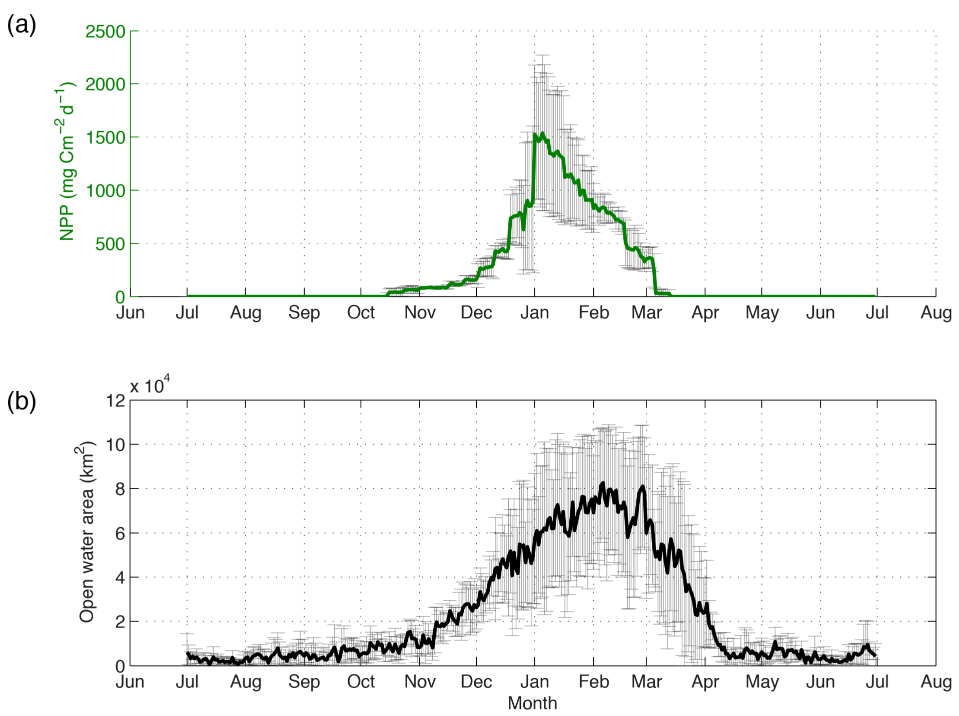

Figure S2. Satellite-based averaged time series of net primary production (NPP) (Fig. S2a) and open water area (Fig. S2b) in the Amundsen Sea coastal polynya between 2010 and 2013. The sea ice images exhibit the spatial extent of the Amundsen Sea coastal polynya, which widens along the Dotson and Getz ice shelves (Fig. S1). Phytoplankton biomass is higher in the center of the coastal polynya than around the edges of the pack-ice boundary and ice shelves[31]. The average primary production of the Amundsen Sea coastal polynya between 2010 and 2013 highlights the clear seasonal variation associated with the timing of opening and closing. The coastal polynya begins to open at approximately 5,000 km^2 in October and peaks at >80,000 km^2 in February, remaining until March. Primary production begins to rapidly increase in November (<50 mg C m^{-2} d^{-1}), with the largest increase between December and January due to the high solar radiation and extensive decline in sea ice concentration. A peak is observed with 1,550 mg C m^{-2} d^{-1} at the beginning of January. Primary production rapidly declines by March after a phytoplankton bloom in January as low as the pre-bloom period, which might be associated with nutrient limitations or increased grazing by zooplankton. This pattern is similar to the temporal variability in phytoplankton biomass and sea ice concentration in the Amundsen Sea coastal polynya over the last decade[31].

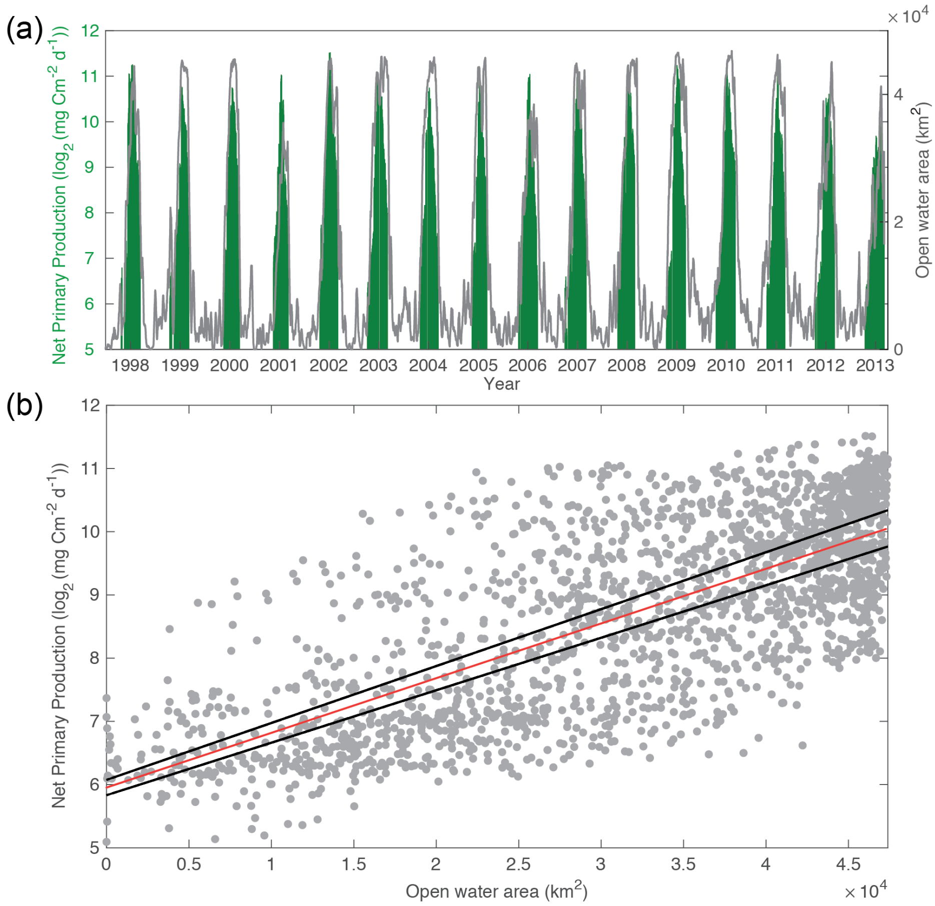

Figure S3. Net primary productivity (NPP) in the open water areas in the Amundsen Sea. a, Time series of the averaged daily NPP and the open water area in the Amundsen polynya (red box) from July 1997 to March 2013. Over the 16-year time series, the NPP was coupled with the open water area due to the seasonal variability of sea ice concentration. NPP and the open water area generally increased from October through March throughout the year, with interannual differences. **b,** Scatter plot of the averaged daily NPP versus the open water area in the Amundsen polynya (r=0.73, p<0.01). The red and black lines represent the linear relationship between the NPP and open water area with 95% confidence limits, respectively. The regression equation is NPP=5.95+8.65e-5 open water area (r^2=0.53, n=5,727). NPP was significantly correlated with the open water area, showing that waters become more productive when the SIC decreases.

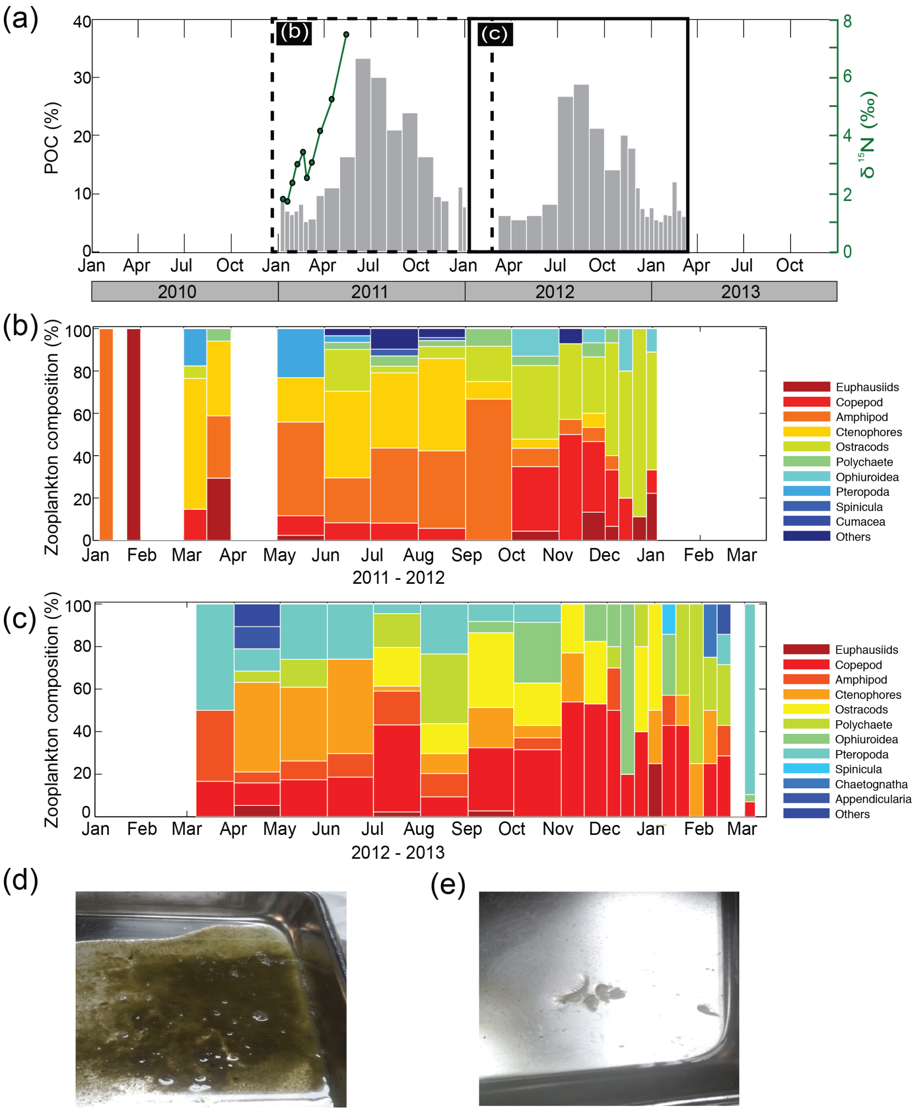

Figure S4. Zooplankton compositions in the sediment trap. a, Particulate organic carbon (POC) and nitrogen isotope[48] among sedimenting materials collected in the sediment trap. **b,** Monthly variations in intact zooplankton compositions from January 2011 to January 2012. **c,** Monthly variations in intact zooplankton compositions from March 2012 to March 2013. **d,** Samples from the sediment trap during February 24–March 4, 2012. **e,** Samples from the sediment trap during July 3–August 3, 2012. The POC in the contribution of carcasses of zooplankton in the sediment trap exhibited a clear seasonal variation showing an increasingly dominant contribution (41−83%) among sedimenting materials between May and October, when the POC fluxes were very low (Fig. 1c). During phytoplankton bloom periods in February and March 2012 and December 2012 and March 2013, diatom and *P. antarctica* colonies (Fig. S4d) are dominant but decrease from March[48]. Phytoplankton colonies were not found after

April[48], whereas the number of swimmers increased between April and October (Fig. S4b and S4c). In this observation, 13 groups of zooplankton (euphausiids, copepods, amphipod, ctenophores, ostracods, polychaete, ophiuroidea, pteropoda, spinicula, cumacea, chaetognatha, appendicularia, and others) were identified from the sediment trap samples. The total number of individual zooplankters collected in the trap clearly shows the seasonal variability during the observation period. During the high organic carbon flux period between January and March (Fig. 1b), the total number of zooplankton is low, but it increases noticeably between April and October and then drops again from October to February of the following year. Sediment traps are designed to collect particles sinking through the water column; thus, it can be difficult to quantitatively observe live zooplankters due to their swimming behavior. Nevertheless, sediment traps can provide an indication of the seasonal distribution of individual zooplankton. Higher zooplankton (swimmers) numbers in autumn/winter than in summer may explain the regular high acoustic backscatter observed at depths >400 m in the moored ADCP from April to November compared to the rest of the year (Fig. 1d).

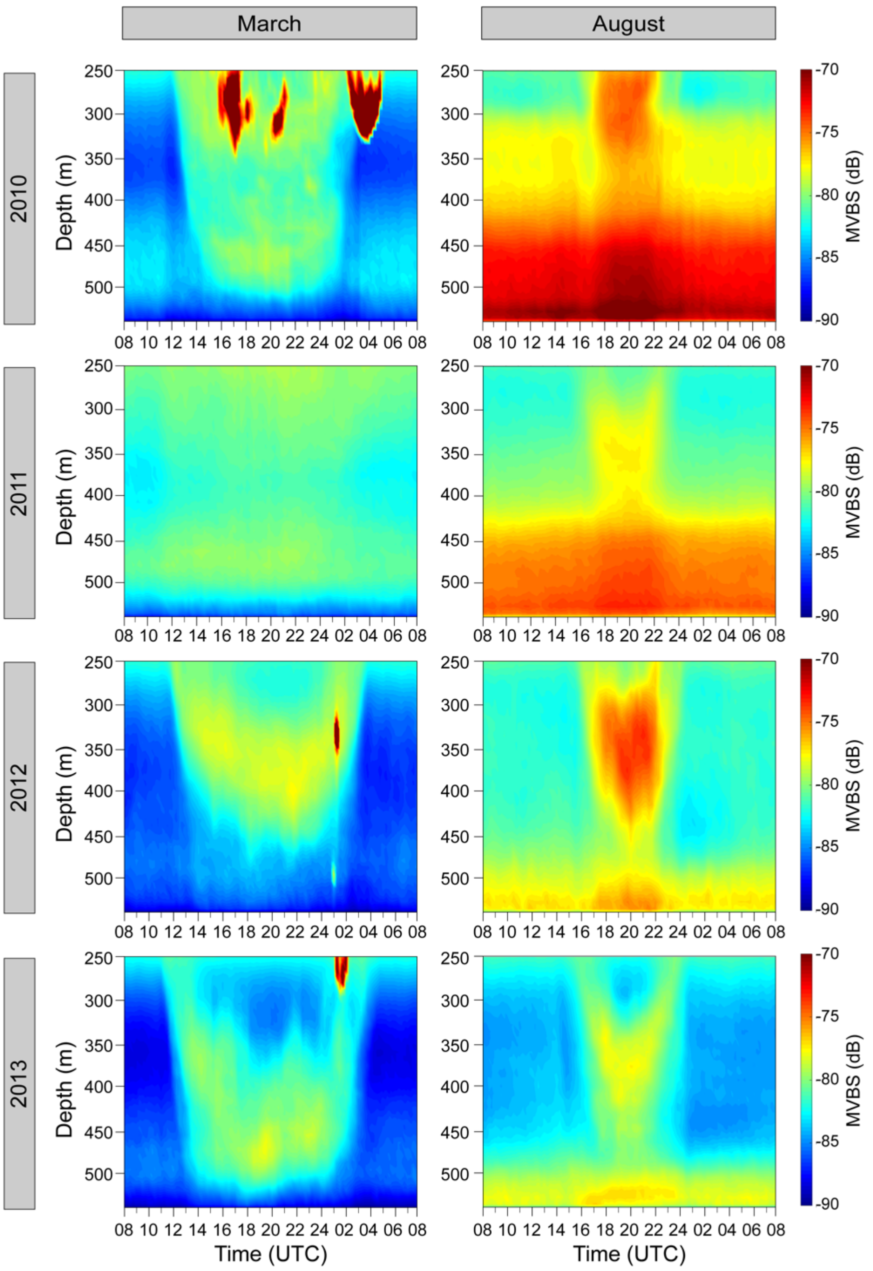

Figure S5. Mean diel variability of zooplankton during late summer (March) and late winter (August). MVBS indicates the mean volume backscattering strength (dB *re* 1 m^{-1}). Clear diel vertical migrations (DVM) of zooplankton were generated in depths of 250–500 m at 11:00–04:00 during summer. In late winter, diel variability of zooplankton was irregularly observed each year while the high MVBS was constantly observed in depths of 400–540 m at all time.

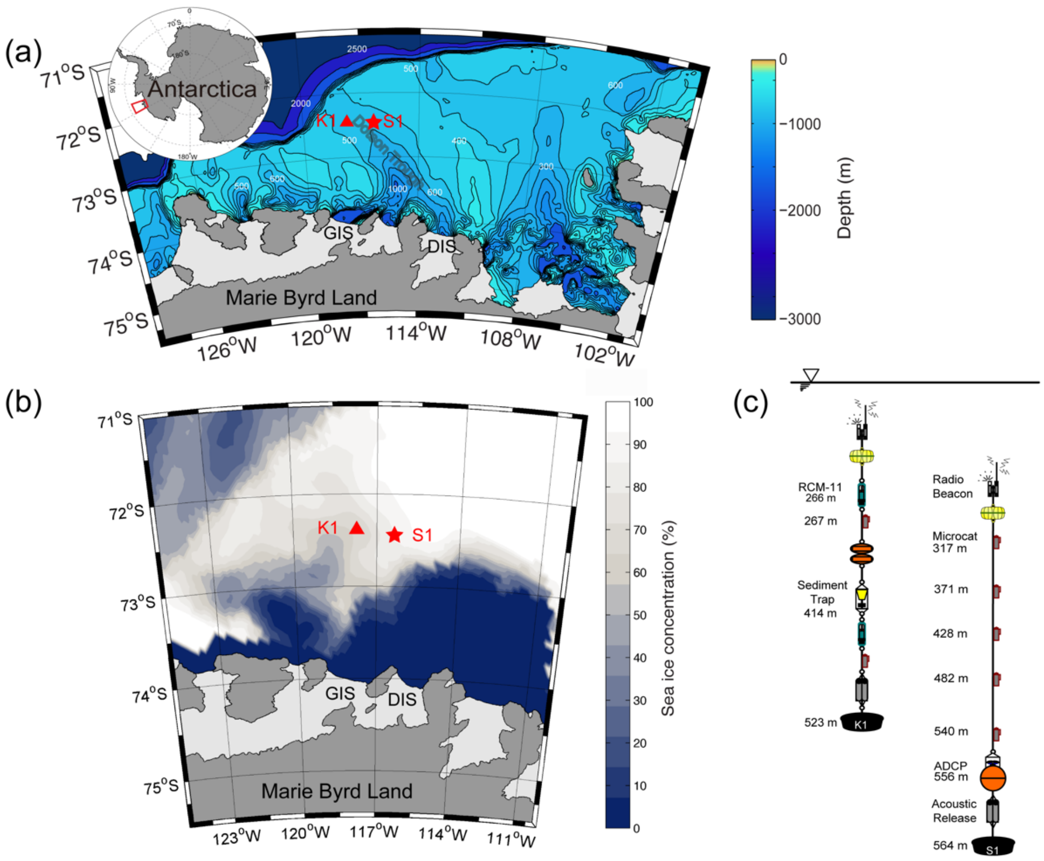

Figure S6. Study area and deployed moorings in the Amundsen Sea. a, The bathymetry is from Nitsche et al.[49], and the black lines are isobaths with 500 m intervals from 0 to 2500 m. The triangle (K1) and star (S1) indicate the respective mooring locations for the acoustic Doppler current profiler (ADCP) and sediment trap. **b**, The mean sea ice concentration (SIC) during January 2011. **c**, Detailed sketches of the subsurface mooring system. In **a-b**, GIS: Getz Ice Shelf; DIS: Dotson Ice Shelf. The Amundsen Sea is considered with a relatively narrow continental shelf, which permits the intrusion of the warm, salty and nutrient-rich Circumpolar Deep Water (CDW) close to the ice shelves[50]. The moorings are located in the Dotson Trough, which is one of main exits for the water column over the Amundsen Sea shelf break. A persistent inflow of the CDW from the deep ocean towards the GIS and DIS[50-52] has been observed[53,54]. Moreover, the moorings are located within seasonally varying sea ice, creating an ideal environmental setting for investigating the seasonal behavior of the zooplankton response under various conditions of surface solar radiation and sea ice.

Table S1. Details of the deployment and recovery of the ADCP (S1) and sediment trap (K1) moorings.

St.	Latitude (S)	Longitude (W)	Deployment date (YYYY/MM/DD)	Recovery date (YYYY/MM/DD)
S1	72° 27.345′	116° 20.327′	2010/02/16	2012/03/01
	72° 27.286′	116° 20.936′	2012/03/02	2014/01/03
K1	72° 24.163′	117° 43.341′	2010/12/31	2012/03/02
	72° 23.208′	117° 46.627′	2012/03/04	2014/01/02

Table S2. Details of cup opening date and sampling intervals of sediment trap.

Cup number	Cup opening date (YYYY/MM/DD)	Time interval (days)	Cup opening date (YYYY/MM/DD)	Time interval (days)
1	2011/01/05	9	2012/03/07	25
2	2011/01/14	9	2012/04/01	30
3	2011/01/23	9	2012/05/01	31
4	2011/02/01	9	2012/06/01	30
5	2011/02/10	9	2012/07/01	31
6	2011/02/01	10	2012/08/01	31
7	2011/03/16	15	2012/09/01	30
8	2011/03/05	16	2012/10/01	31
9	2011/04/05	30	2012/11/01	15
10	2011/05/05	31	2012/11/16	15
11	2011/06/05	30	2012/12/01	9
12	2011/07/05	31	2012/12/10	9
13	2011/08/05	31	2012/12/19	9
14	2011/09/05	30	2012/12/28	9
15	2011/10/05	31	2012/01/06	9
16	2011/11/05	15	2012/01/15	9
17	2011/11/05	15	2013/01/24	9
18	2011/12/05	9	2013/02/02	9
19	2011/12/05	9	2013/02/11	9
20	2011/12/05	9	2013/02/20	9
21	2011/12/05	8	2013/03/01	15

References

47. Tamura, T., Ohshima, K. I., Fraser, A. D. & Williams, G. D. Sea ice production variability in Antarctic coastal polynyas. *J. Geophys. Res. Oceans* **121,** 2967–2979, https://doi.org/10.1002/2015JC011537 (2016).

48. Kim, M. *et al.* Sinking particle flux in the sea ice zone of the Amundsen shelf, Antarctica. *Deep Sea Res. Part I: Oceanogr. Res. Papers* **101,** 110–117, https://doi.org/10.1016/j.dsr.2015.04.002 (2015).

49. Nitsche, F. O., Jacobs, S. S., Larter, R. D. & Gohl, K. Bathymetry of the Amundsen Sea continental shelf: implications for geology, oceanography, and glaciology. *Geochem. Geophys. Geosyst.* **8,** 1–10, https://doi.org/10.1029/2007GC001694 (2007).

50. Wåhlin, A. K., Yuan, X., Björk, G. & Nohr, C. Inflow of warm circumpolar deep water in the central Amundsen shelf. *J. Phys. Oceanogr.* **40,** 1427–1434, https://doi.org/10.1175/2010JPO4431.1 (2010).

51. Wåhlin, A. K. *et al.* Variability of warm deep water inflow in a submarine trough on the Amundsen Sea Shelf. *J. Phys. Oceanogr.* **43,** 2054–2070, https://doi.org/10.1175/JPO-D-12-0157.1 (2013).

52. Ha, H. K. *et al.* Circulation and modification of warm deep water on the central Amundsen shelf. *J. Phys. Oceanogr.* **44,** 1493–1501, https://doi.org/10.1175/JPO-D-13-0240.1 (2014).

53. Jacobs, S. S., Jenkins, A., Giulivi, C. F. & Dutrieux, P. Stronger ocean circulation and increased melting under Pine Island Glacier ice shelf. *Nat. Geosci.* **4,** 519–523, https://doi.org/10.1038/ngeo1188 (2011).

54. Jenkins, A. *et al.* Observations beneath Pine Island Glacier in west Antarctica and implications for its retreat. *Nat. Geosci.* **3,** 468–472, https://doi.org/10.1038/ngeo890 (2010).
